# Being on the juvenile dermatomyositis rollercoaster: a qualitative study

**DOI:** 10.1186/s12969-019-0332-7

**Published:** 2019-06-18

**Authors:** Polly Livermore, Suzanne Gray, Kathleen Mulligan, Jennifer N. Stinson, Lucy R. Wedderburn, Faith Gibson

**Affiliations:** 1 University College London Great Ormond Street Institute of Child Health, London, UK; 2 Centre for Outcomes and Experience Research in Children’s Health, Illness and Disability (ORCHID), Great Ormond Street Hospital for Children NHS Foundation Trust, London, UK; 3 Evelina London Children’s Hospital, GSTT NHS Foundation Trust, London, UK; 4 City, University of London, London, UK; 5 East London NHS Foundation Trust, London, UK; 6 Hospital for Sick Children, Toronto, Canada; 7 Lawrence S. Bloomberg Faculty of Nursing, University of Toronto, Toronto, Canada; 8 NIHR Biomedical Research Centre at Great Ormond Street Hospital, London, UK; 9 Centre for Adolescent Rheumatology Versus Arthritis at UCL UCLH and GOSH, London, UK; 10 School of Health Sciences, University of Surrey, Surrey, UK; 11 NIHR Clinical Doctoral Research Nursing Fellow, Infection, Immunity and Inflammation, 6th Floor, Institute of Child Health, University College London Great Ormond Street Institute of Child Health, 30 Guilford Street, London, WC1N 1EH UK

**Keywords:** Juvenile dermatomyositis, Qualitative research, Phenomenology, Uncertainty, Psychosocial needs

## Abstract

**Background:**

Juvenile Dermatomyositis is a rare, potentially life-threatening condition with no known cure. There is no published literature capturing how children and young people feel about their condition, from their perspective. This study was therefore unique in that it asked children and young people what is it like to live with Juvenile Dermatomyositis.

**Methods:**

Data were obtained from fifteen young people with Juvenile Dermatomyositis, between eight and nineteen years of age from one Paediatric Rheumatology department using audio-recorded interpretive phenomenology interviews. Data were analyzed phenomenologically, using a process that derives narratives from transcripts resulting in a collective composite of participants shared experiences, called a ‘phenomenon’.

**Results:**

The overarching metaphor of a rollercoaster captures the phenomenon of living with Juvenile Dermatomyositis as a young person, with the ups and downs at different time points clearly described by those interviewed. The five themes plotted on the rollercoaster, began with confusion; followed by feeling different, being sick, steroidal and scared from the medications; uncertainty; and then ended with acceptance of the disease over time.

**Conclusion:**

Young people were able to talk about their experiences about having Juvenile Dermatomyositis. Our findings will aid clinicians in their practice by gaining a deeper understanding of what daily life is like and highlighting ways to enhance psychosocial functioning. Hopefully, this study and any further resulting studies, will raise understanding of Juvenile Dermatomyositis worldwide and will encourage health care professionals to better assess psychosocial needs in the future.

## Background

Juvenile Dermatomyositis (JDM) is a rare, potentially life threatening, systemic condition of unknown origin, characterized by weakness in proximal muscles and skin rashes, often involving other systems [1–4]. Weakness is progressive, which can first become evident with having difficulty climbing stairs and can become profound, with children progressing to becoming bed bound, unable to sit up or roll over and for some, they may even require nutritional support through feeding tubes [5–7]. In severe cases, this debilitating condition involves children and young people (CYP) requiring multiple hospitalizations, experiencing extreme muscle pain, requiring daily physiotherapy and cytotoxic medications to gain symptom control, and as a result they miss months of schooling [8–10].

The term ‘psychosocial’, pertaining to both psychological and social, encompasses a multitude of variables, such as social and emotional support, mood and anxiety. Research into psychosocial needs in CYP with JDM is sparse. One of only two published studies examining Quality of Life (QoL) in JDM found significantly lower health related QoL in this group compared to controls [11]. The study asked parents of 272 children with JDM to report their child’s QoL using the Childhood Health Assessment Questionnaire (C-HAQ). Baseline disability and longer disease duration were major determinants for poor well-being at follow up [11]. More recently, in a study exploring the psychosocial impact of having a child with JDM, 39 caregivers were asked to complete questionnaires. Results indicate that children with active JDM had significant lower QoL and family functioning compared to normative populations [12]. A major limitation of both these studies was the exclusion of CYP as research participants. The ‘proxy problem’ of whether parental proxy reports corroborate with child self-reports examining QoL has been well described in the literature [13–15], with the consensus that parents generally underestimate the impact of health-related QoL. These studies highlight the need to ask children and young people to report their own QoL and psychosocial needs. In addition, rather than examining psychosocial needs from all dimensions, QoL is the main focus reported in the literature following physical health, missing all the other domains of living with a chronic condition, such as schooling and emotional adjustment.

Given these gaps in our understanding of the perspectives of CYP with JDM, this study has much to contribute. The research question this study sought to answer was: “What is it like to have JDM?”

## Study design

### Methodology

Phenomenology is both a philosophy and a research methodology concerned with the ‘lived world’ [16], comprising different variations and interpretations, predominantly under two broad headings of Descriptive or Interpretive. Descriptive phenomenology is guided by the work of Husserl, its aims are to describe a phenomenon’s general characteristic rather than the individuals experiences [17, 18]. Interpretive phenomenology supported by Heidegger, aims to describe, understand and interpret phenomenology, put simply as moving from description to interpretation [17, 19]. Interpretive phenomenology was the methodology of choice for this qualitative study as the researcher sought to understand the human experience [20]. This study asked young people to tell their story of the experience of having JDM, the researcher then interpreted these stories to uncover meaning, in order to bring the phenomenon into the open [21].

### Sample and setting

Children and young people were identified from clinic lists and prospective ward admissions at a specialist tertiary hospital in London, United Kingdom. The inclusion criteria was any child between the ages of eight and nineteen years of age with a diagnosis of JDM; only excluding CYP with any confounding medical condition which may alter their psychosocial needs, such as diabetes, however, of note none were excluded.

## Methods

Open-ended unstructured interviews allowing participants to share their experience were used; the method of choice for phenomenology. However, there is limited research using phenomenology with CYP as there are perceived inherent difficulties in encouraging CYP to freely express their perceptions of their lived experience without inadvertently directing them. Therefore, participants were also given the option to use creative methods in the interview to help them tell their story. Three techniques were used: (1) Body mapping; predominantly used to illustrate location of pain [22–24], however, due to its multifaceted nature it allowed CYP to draw or write their interpretation of how their JDM affects them; (2) Time-lining; useful to help CYP tell their journey and allow the participant to decide how much to reveal [25, 26]; and (3) Comic book design; this was used to help CYP tell their story in their own style and time [27–30]. Each of these techniques allowed CYP to reveal their experience from their developmental understanding and thus reducing the power imbalance, the need for eye contact and providing a buffer when discussing sensitive issues such as their feelings about their JDM.

### Data collection

Potential participants were approached by a member of the clinical team when they attended routine clinic appointments. The first seventeen CYP who were seen in clinic and met the inclusion criteria were approached to take part in the study. Parents of two CYP, did not want to take part when approached, due to work commitments; the remaining fifteen were included in the study. Recruitment was easier than anticipated as there were minimal exclusion criteria, and families were eager to participate. Families chose whether to be interviewed in the hospital or at home; two parents asked to be present for the interviews, but they remained in the background. The audio-recorded interviews were carried out by the lead researcher (PL). Ten out of fifteen interviewees, especially the older participants, said they preferred to talk rather than use creative methods, thus the interview style was an open-ended, unstructured interview, beginning with ‘tell me about your JDM’. The interviews took place between August and December 2017.

### Data analysis

**Fig. 1:**
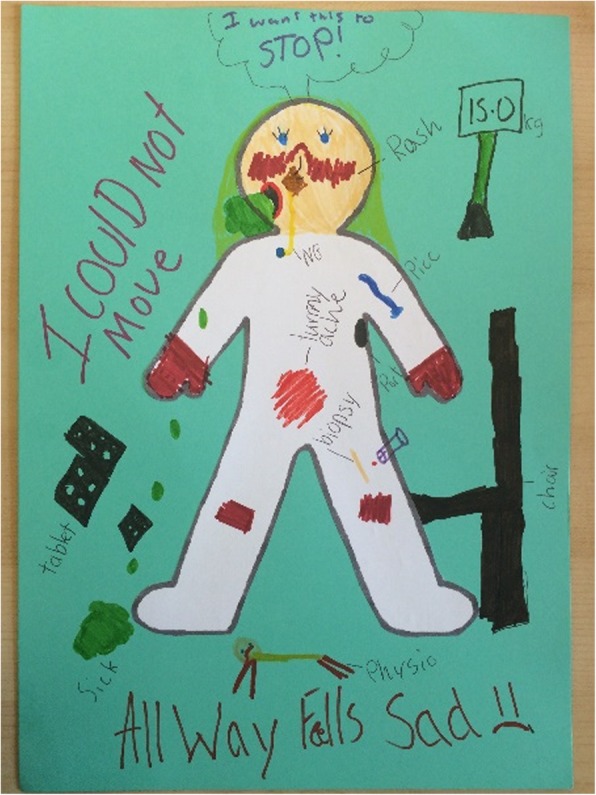
A drawing representing participant’s experience of Juvenile Dermatomyositis. Children and young people were given the option of drawing how they view the effects of Juvenile Dermatomyositis on their body, either as part of the interview (a warm up activity) or if they did not want to talk, instead of the ‘standard interview’. They were offered a blank ‘bodymap’ and encouraged to complete however they wanted to, whether in text or drawing. The young person who completed this example, explained that they were drawing; the rash on the hands and face and knees, the biopsy site, the intravenous line in situ, constant tummy ache, vomit due to the medicines, tablet packets, scales (as they had lost so much weight) and a chair which they required as they couldn’t stand. They went on to write on their picture “I could not move”, “Always feel sad” and a thought bubble showing “I want this to STOP”

Audio-recordings were transcribed verbatim by the lead researcher. The average interview length was an hour, ranging from 18 min to the longest being 130 min. One CYP drew their timeline and one drew in the comic book, with the same aim of describing their journey with JDM. Three children drew the effects of their disease upon the body-map outline, see Fig. 1 for an example from a ten year old. The visual methods were checked alongside the transcribed interviews to ensure all written and drawn creations were mentioned verbally.

The aim of interpretive phenomenology is to describe, understand and interpret experience from those experiencing it [17, 19, 31], achieved through a circular continuous process of reading, writing and talking [32]. A method proposed to derive narratives from transcripts [33] was modified into clear steps (Table 1). This process begins with the reading and re-reading of the transcripts, before removing repetition and superfluous text. As the process continues; words are added to make the text readable and finally crafted keeping the data true to its original meaning whilst showing what the researcher is interpreting. In this study, this resulted in fifteen narratives, one from each participant. These were then plotted alongside each other looking for shared meaning within each of the voices. Subsequently, an overarching phenomena which captured living with JDM was identified with five shared themes within it, and these themes were then plotted together as separate narratives to complete the data analysis. The themes were reviewed as they developed by both the lead researcher PL and doctoral supervisor FG: data saturation was felt to be reached at fifteen interviews.

Rigour and trustworthiness were enhanced by the lead researcher keeping a reflexive diary, maintaining a clear audit trail, undertaking a presuppositions interview and the transcripts and final results were read by two members of the supervisory team with constant discussion. Two members of the supervisory team are psychologists, and thus were invaluable in directing, assisting and supporting the lead researcher throughout the study. It is worth noting that phenomenology has been critiqued for its inherent bias, however the findings presented are always simply the impressions gained, as it presents someone else’s lived experience [31].

Phenomenology often takes the end results back to the individual participants to ‘member check’, however, there was concern that this could cause upset in the young people when reflecting upon their thoughts about living with a chronic condition. Therefore, as a compromise, after data analysis had occurred, it was decided to invite the young people back as a group to discuss general themes, all completely anonymised.

## Results

Fifteen young people participated in this study (Table 2). In keeping with phenomenology, characteristics such as economic status and education level were not collected as the focus was on individuals own experience of their unique journey. Individual patient data reported were kept to a minimum to protect participant anonymity in such a rare disease. However, two thirds of the young people interviewed had a chronic disease course lasting longer than five years in length. The sample was heterogeneous: length of disease (from three weeks to sixteen years); severity of disease (from acute inpatients to those in remission off medication); treatments received; and knowledge and support. This diversity was apparent when reading the narratives, however, all progressed through similar stages at similar times of their disease course.

**Table 1:** Steps of data analysis taken when conducting interpretive phenomenology, adapted from Caelli [33]

1. Reading and re reading whole interview many times	
2. Highlighting the first level extra text which can come out e.g. interviewer questions or listening noises where not important, and ‘umms’ and ‘huh’ which are extra	
3. With first level text removed	
4. Second level coding procedure to identify sections that wander away from phenomena, are superfluous e.g. when discussing what they had for lunch or do not make good grammatical English	
5. With second level text removed	
6. Third level, all of it with added words to make it readable, but keeping participant words wherever possible	
7. Craft into story, in a way that ‘shows’ what the researcher is noticing and interpreting whilst working with the data and keeping it true to original meaning	
8. With comments added to show initial themes of each paragraph of crafted story

**Table 2:** Demographic details of study participants

Gender	6 males, 9 females
Ethnicity	12 White British, 3 other
Age at interview	8–18 years (median 12 years, 7 were 8-12 yrs., 8 were 13-19 yrs)
Age diagnosed	2–16 years (median 8 years)
Disease duration	3 weeks – 16 years (median 5 years)
Disease severity	From acutely unwell in-patients, to in remission, off medicines
Disease course	10 chronic, 4 monocyclic, 1 polycyclic
Medications received	15 had received Methotrexate, 7 had Anti-TNF Treatment

**Fig. 2:**
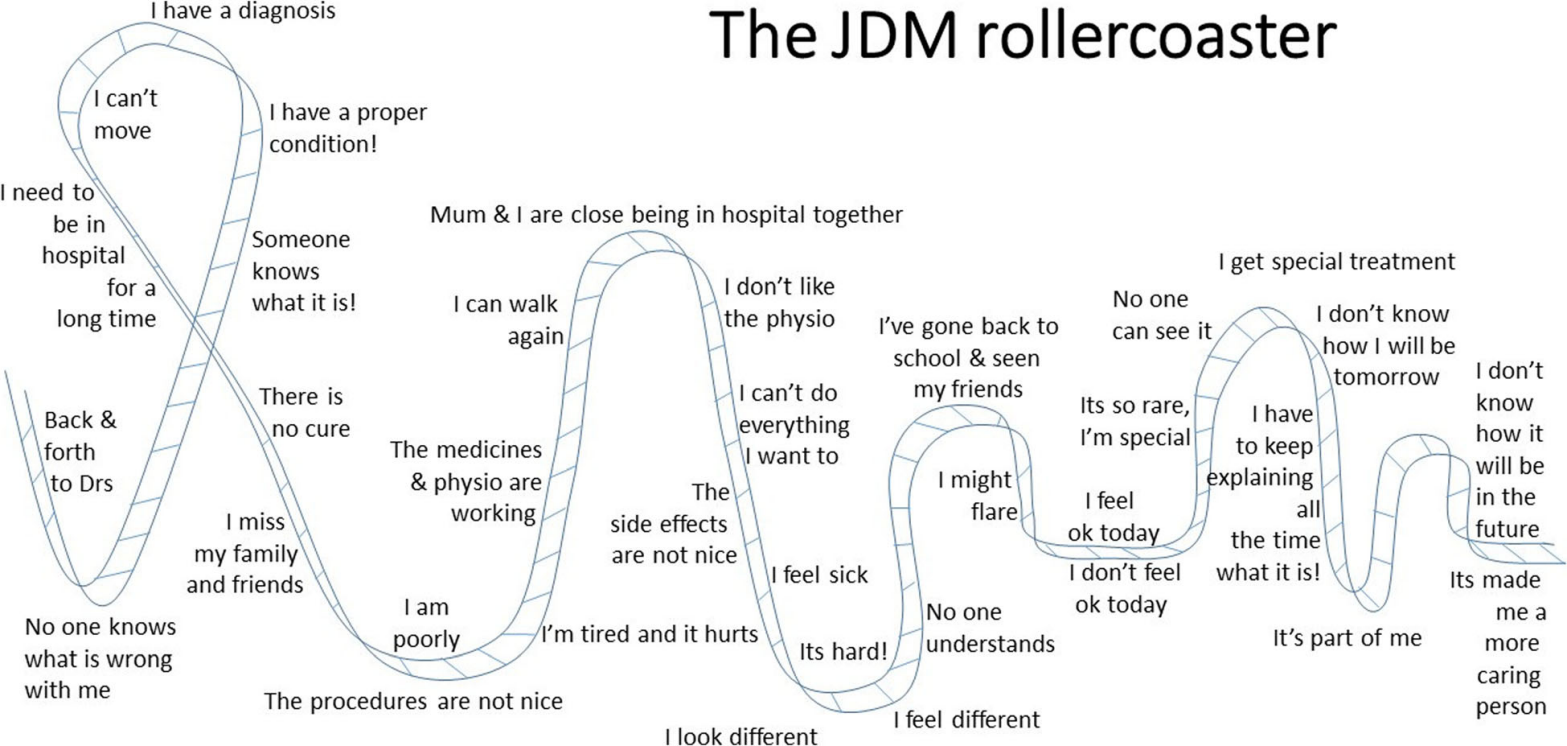
The illustrated Rollercoaster Metaphor. This visual creation was developed after considering the journey which young people were describing. The downward track in the beginning as they began to get unwell from the Juvenile Dermatomyositis and the confusion from not knowing what is wrong. Then some relief and a journey up the rollercoaster as they finally get a diagnosis and a name for their symptoms, something which they can research and tell family and friends. This is followed back down the track as the realisation that it can be a serious condition, often at this stage accompanied by active, painful disease as they wait for treatment therapies to begin. Then back up the track as the treatments have started and they begin to feel better, maybe become able to move more and regain some strength. The rollercoaster continues like this as each young person described the seesaw of emotions, feelings and symptoms as they travelled on their JDM journey. The rollercoaster ride can go on for many years, with young people having ups, downs and plateaus from day to day and month to month, even year to year. This depiction can be used as it is shown, or modified to meets the needs of individual children, young people and their families in clinic to illustrate the rollercoaster and to help alleviate some of the unpredictability, confusion and increase their knowledge and understanding; preparing them for the future

The researcher (PL) was immediately struck by the magnitude of the impact of JDM on the equilibrium of the CYP. To a young child or adolescent who is leading a normal, healthy and active life; to be suddenly knocked off balance, almost by something out of nowhere, something which potentially renders you unable to move, was hugely significant for all of the participants. It was this impact, and how this was experienced by individuals that was being verbalised in these stories. It was more than just knocking them over, it was the repetition, doing it again and again, taking them up and dropping them down. Hence the emergence during data analysis of a metaphor of a rollercoaster, helped to illustrate this experience visually. It was then possible to describe all fifteen stories using this metaphor. For example, the youngest and very recently diagnosed hospitalised child in the study had just stepped onto the rollercoaster, experiencing the first bottom dip, whereas the eighteen year old who had been diagnosed at two years of age, had come so far on the rollercoaster ride that now they could not remember what came before. This overarching phenomena of the *rollercoaster*, with the ups and downs associated with the common emotions, fears and challenges, was a typical journey for all those interviewed and could be illustrated visually when describing a journey with JDM (Fig. 2). Within the overarching phenomena of the rollercoaster there were ‘themes’, almost encapsulating each step along the way. The themes expressed as described by the CYP included *confusion; feeling different; sick, steroidal and scared; uncertainty; and acceptance*.

Each of these five themes is presented in turn and illustrated using a combination of voices from the shared stories, consequently, unlike other types of qualitative research, the quotes cannot be identified from one individual as some may be from more than one participant. This anonymity is also essential for the families when sharing the sensitive words of few young people with a rare condition.

### Confusion

Young people talked about *confusion* for different reasons. Even before a diagnosis, participants talked overwhelmingly about this confusion that the JDM seemed to come out of nowhere and affected them in ways that they found confusing, especially the impact of losing muscle strength;

“We saw so many doctors, I remember just being so anxious about what was going on, I didn’t know why my body wasn’t functioning in the way it should.”

Other reasons mentioned, included confusion that no one knows what is wrong with them; the confusion that they may be unwell but equally just be considered lazy; the confusion health care professionals have in diagnosing them; and the confusion from their family in trying to support them. Also, the fear from having a rare, often unknown condition which required referrals to other specialist centres was mentioned by a third of the young people. These are exemplified by the following quotes:

“My GP (*General Practitioner*) said he didn’t know what was happening to me, so he referred me to my local hospital, but they didn’t know what was wrong with me.”

One of the young people talked about the speed of onset in her particular case:

“At the beginning, I just started as a normal 10 year old being able to do cross country with my class and then suddenly I woke up and I wasn't able to do anything and I think it’s how quickly it happens.”

Confusion also occurred when there was a lack of understanding, amplified if those around you, for example health care professionals, teachers and parents are also confused, and this was most apparent at diagnosis and hence placed at the beginning of the rollercoaster.

### Feeling different

*Feeling different* was striking when reading all the 15 separate narratives. Whilst ‘feeling different’ spanned the whole rollercoaster, there were specific times when it was particularly apparent, such as in the beginning when the young people developed the vivid facial rash as described here by the following quote:

“I hated the way that I looked so I wouldn’t leave the house without make up on. My face was literally bright red and swollen, and I just looked horrible, like a baboons bum.”

All of the CYP who had JDM diagnosed at a younger age raised the noticeable differences that it brings in the beginning, such as the facial rash and gottrons papules; but all highlighted that as the disease progressed these often fade and become difficult to see. The majority of the young people thought this was a negative thing as they have to keep explaining what is wrong with them;

“JDM is hidden, it’s more inside the body, it’s not like a visible disability with a wheelchair all the time, so just because you can’t see it, it doesn’t mean it’s not there.”

The CYP articulated the need to explain what JDM is and how this makes them different to their peers;

“People don't know what it’s like at all and they've never heard of the condition before so I have to explain the same thing 24/7 and it's really irritating to explain all the time and for no one to understand at all.”

Within this theme, there was also an expression of clear advantages to being different such as receiving treats and nominations towards special trips. As one said;

“It turned out that having the illness has good moments as well because it’s so rare people took an interest. I got invited to this party for people with bad illnesses, so you do feel kind of special.”

### Sick, steroidal and scared

Captured in this theme was the concerns expressed by young people when discussing their medications they had to take, and the resulting effects. One teenager used the term “steroidal” specifically when talking about the effects caused by the steroids; others openly talked about their nausea and sickness caused by the methotrexate; the ‘scared’ emotion was expressed when describing the methotrexate injections and not knowing how they might feel during the subsequent days, often anticipating the worst.

The effects of the medications were mentioned always in a negative manner, especially methotrexate as exemplified by this quote:

“I’d feel sick before I even had it, then I started almost throwing up when I was having the injection, and having a panic attack before it because the day afterwards, no matter what I did, I would feel sick, and just wanting to cry because I just felt so horrible.”

Fear from the methotrexate; both unpredictable side effects and the needles themselves were discussed;

“My mum would bring it in, I’d sit there being like, ‘No,’ shaking, and she’d swipe my leg (*with an alcohol wipe*), and then I’d literally be grabbing onto her arm, and she’d have to just do it as quickly as she could, and I’d just cry. So this became a routine thing on a Friday, which is obviously not the best way to spend your Friday nights. All my friends are out, and I’m sitting in bed crying.”

Steroids are known to cause weight gain, stretch marks and a cushingoid face. They are often used in JDM to reduce inflammation in the beginning and at times of disease flare, and especially to the adolescents in this study; they had a huge psychosocial impact that featured in their stories;

“My appearance really changed as I noticed my face went very red at the time, I had the round chubby moon face, most of my body put weight on and I became hairier. I was thinking, ‘I’m getting uglier.’”

### Uncertainty

All the young people talked very clearly about *uncertainty* about their JDM, not only their own uncertainty but also that of others;

“The teachers told them I'm allergic to the sun which isn't actually true, because she doesn’t understand either, I don’t think any of the school teachers know about it.”

There was uncertainty about why they were unwell, uncertainty with the day-to-day effects, the relapsing and remitting nature of the disease, uncertainty about the future and about the effects JDM had on them, as this quote highlights;

“When I went home from the hospital, my parents moved all my stuff downstairs so that my room was in what was my dad's office. You know the feeling when you're on a holiday but it is raining and you feel a bit miserable? It was like that because you didn’t know what was happening. Everything had literally just been thrown off balance and it was scary.”

For example, one of the ten year olds in the study, articulated that because there was no cure, no one can know what is going to happen, again highlighting the impact uncertainty can have.

### Acceptance

During the interviews, half of the CYP expressed ‘a degree of needing to get on with life’, and as one described it, ‘accept JDM for what it is, because you can’t change it’. Whilst the majority who talked more frequently about accepting their condition, had by their own admission ‘less active JDM now’, even those who were in hospital for treatment or experiencing disease flares, had a level of maturity and degree of acceptance to their disease as this thirteen year old explains;

“I still say to myself that I'm going to get better either way, like it may take longer than other people, may be shorter or just the same, but I know eventually I will get better.”

Some young people talked about how they feel their JDM has changed their view on life, as this quote illustrates;

“My JDM has made me a better person, I used to be very negative and now I'm positive. I embrace my JDM and I feel more powerful that I have it, I just feel good that I had it as it has taught me a lot of things.”

Resilience was evident within the discussions; whilst CYP openly talked about how things have been hard, they equally wanted to share the importance of getting on with life;

“You get over it, because you don’t have a choice, that’s the thing that I learnt fairly quickly, that it’s fine to be sad about it, but if you’re going to sit there and be sad about it, you’re going to let it stop you living your life, because it’s not going to go away.”

## Discussion

This study invited children and young people to share their experience of living with JDM. Those participating had a range of disease durations, disease severities and unique experiences. However, there were marked similarities in the stories they told and this was captured through the phenomenon of a rollercoaster. *Feeling different*, *sick, steroidal and scared* and having *uncertainty* were themes which spanned the whole of the rollercoaster, but would be more prominent at different time points depending on what else was happening to the young person in their life; whether due to their JDM, such as disease flares, or socially, such as changing schools. There was also some overlap between these themes. For example a statement on the effects of medications, could equally fit into *feeling different*, or *sick, steroidal and scared*. However, the rollercoaster does not imply a linear process ending in acceptance. The six CYP who were interviewed and who later attended a follow up session to hear and discuss the themes and rollercoaster metaphor, agreed that acceptance of JDM and the limitations and challenges it brings is a helpful place to get too, but may not be sustainable or appropriate for all children and young people.

The ups and downs which illustrated the journey CYP with JDM talked about, have obvious commonalities with other chronic illnesses. The rollercoaster metaphor has been used previously in the literature to describe other health events such as people acquiring a physical impairment in youth ‘The Rollercoaster Ride’ [34], becoming the parent of a deaf child ‘The roller-coaster of experiences’ [35], and ‘Rollercoaster Asthma’ [36]. Looking specifically at other paediatric auto-immune conditions, the study by Gomez-Ramirez (2016) describes parents journeys when caring for their children with juvenile arthritis as akin to the recurring ups and downs of rollercoaster rides, with parents feeling an underlying anxiety [37], which mirrored some findings in this study. A further study in juvenile arthritis, this time in the young people themselves, report similar themes, such as those of aversion to being different and suspension in uncertainty. However, there are JDM specific elements which have emerged out of this study, and whilst they individually have similarities with other health conditions, the impact of putting these together constitutes the lived experience of JDM. As evidenced through the quotes, these included the difficulty in diagnosis due to its rarity; the speed of onset; the impact of losing muscle strength and potential inability to move; the constant need to explain what the condition is; the invisibility over time; the remitting nature; and lack of a cure. Due to the lack of research, it is not possible yet to compare the psychological impact of JDM compared to other chronic illnesses, but this research is ongoing.

Juvenile Dermatomyositis is a rare condition typically managed in specialist centres around the world. The physical effects of JDM are often presented in the literature [4, 38–40], but this study brings attention to the psychosocial issues. Any ‘tools’ which health professionals employ to measure physical disease status only capture half the picture of how CYP with JDM are really doing. Currently there are no instruments available specifically to assess psychosocial needs of those with JDM, however work is ongoing in this area and the development and dissemination of these will be very valuable to the speciality. Not all treatment centres will have a psychologist or specialist nurse to support young people in their day to day management of their condition, therefore, having an understanding of the psychosocial effects upon CYP of chronic disease in general, and more specifically of JDM, is important for all. Screening for concerns, such as low mood and anxiety is a good place to start and has been shown by Knight (2018) to be vital in young people with Lupus, a similar rheumatic condition [41]. Through providing more developmentally appropriate information on a regular basis and providing an opportunity for young people to voice their anxieties is essential. Furthermore, providing strategies to help CYP manage the inevitable uncertainty of living with an unpredictable chronic condition; ensuring they are assisted to explain their condition in a clear and concise way to their peers and frequent information is given to schools, is recommended. The young people interviewed talked about a lack of knowledge and understanding from their teachers about JDM, and whilst this could apply to other rheumatic conditions, we believe there is no published literature for health care professionals or parents to provide to schools, unlike other conditions such as Juvenile Idiopathic Arthritis (JIA). Whilst there is limited research within the JDM speciality field, there is extensive progress made through over conditions such as oncology, but also within similar autoimmune conditions like juvenile arthritis. For example, these areas of need could be filled by provision of targeted psychosocial interventions, which could include one-to-one counselling or peer support disseminated through a peer mentoring system as described by Kohut et al. (2018) for juvenile arthritis [42].

Having an understanding of the rollercoaster as a metaphor in clinical practice could be helpful to aid clinicians when talking to the CYP and their family about where the young person feels they are. For example, a young child who is struggling, may be able to point at the bottom of the rollercoaster without the need to articulate verbally how they are feeling. Similarly, it can help physicians to discuss certain emotions at more appropriate time points in their care, such as not talking about acceptance, when they are still struggling with confusion. Ultimately, the simplicity of the rollercoaster metaphor is one which can be used clinically to highlight to CYP and their families the turbulent nature of their disease and the path they may follow, whilst scaffolding the specific requirements for CYP to get them to the top of the track, thus reducing some of the inherent confusion around having JDM.

### Limitations

Qualitative studies are small in number of participants, and rich in depth. These CYP were all recruited through one institution and therefore their stories may be different to others who experience other treatments or comorbidities. Asking CYP to talk openly about sensitive topics such as their health, is known to be challenging, but through the addition of creative methods in this study, this made it easier for some. In phenomenology studies, demographic diversity is not prioritised, instead the purpose is to bring various voices to the study, not to compare difference [43]; however, although low, there was a range of disease duration, severity, treatments and age of participants. Whilst the rollercoaster metaphor is likely to be ongoing and adaptive to changes over time, unfortunately due to the cross sectional design of the study this cannot be explored further, longitudinal research is therefore required.

## Conclusions

Better assessment of mental and psychosocial health in children and young people is firmly on the political agenda for both Europe [44] and England [45], however, this is the first study to ask CYP what it is like to live with JDM and thus provides a starting point for health care professionals to work from. Further research is underway to establish whether the concerns raised in this study are typical of the severity of patients we see in this tertiary centre or indeed whether they are shared with a larger UK wide population of CYP with JDM. Together the results of these two linked studies have the potential to improve practice by developing targeted interventions to address psychosocial need in the future. After all, as the CURE JM website states “Whether the course of the disease is mild or severe, Juvenile Myositis is life changing for all of these children and their families” [46].

## Data Availability

The datasets (written interview transcripts) analyzed during the current study are not publicly available as JDM is a rare disease and therefore there is the risk of patient identification from the transcripts but are available from the corresponding author on reasonable request.

## Abbreviations

CHQ: Childhood health questionnaire
CYP: Children and young people
JDM: Juvenile Dermatomyositis
JIA: Juvenile Idiopathic Arthritis
QoL: Quality of Life
